# Integrating Functional Genomic Screens and Multi-Omics Data to Construct a Prognostic Model for Lung Adenocarcinoma and Validating SPC25

**DOI:** 10.3390/cancers17233844

**Published:** 2025-11-29

**Authors:** Yang Zhang, Huijun Tan, Depeng Jiang

**Affiliations:** 1 The Second Clinical College of Chongqing Medical University, Chongqing 400010, China; 2023110697@stu.cqmu.edu.cn (Y.Z.); 2023110686@stu.cqmu.edu.cn (H.T.); 2 Key Laboratory of Respiratory Inflammatory Injury and Precision Diagnosis and Treatment, Chongqing Municipal Health Commission, Chongqing 400010, China

**Keywords:** lung adenocarcinoma, CRISPR-Cas9 screening, prognostic signature model, SPC25, immunotherapy

## Abstract

**Background:** Lung adenocarcinoma (LUAD) presents a significant clinical challenge due to its high heterogeneity and limited treatment efficacy, creating an urgent need for reliable prognostic biomarkers and novel therapeutic targets. Integrating functional genomic vulnerabilities with patient multi-omics data offers a promising approach. **Methods:** We identified genes essential for LUAD cell proliferation from genome-scale CRISPR-Cas9 screening data (DepMap). These were integrated with transcriptomic data from the TCGA-LUAD cohort to select candidate genes. A prognostic risk-score model was constructed using LASSO and multivariate Cox regression analyses and validated in independent GEO datasets. We analyzed the model’s association with clinical features, signaling pathways, tumor immune microenvironment, and drug sensitivity. The predictive value for immunotherapy response was assessed using a real-world cohort. The core gene SPC25 was further validated through in vitro and in vivo experiments and single-cell RNA-seq analysis. **Results:** A robust 7-gene risk-score model was established. This model effectively stratified patient prognosis in training and validation sets and was an independent prognostic factor. A high-risk score correlated with advanced tumor stage and an immunosuppressive microenvironment. High expression of the signature genes predicted poor immunotherapy response. Functional experiments confirmed that SPC25 knockdown significantly inhibited LUAD cell proliferation, migration, and colony formation. Critically, in vivo xenograft experiments demonstrated that SPC25 depletion markedly suppressed tumor growth. Single-cell sequencing revealed high SPC25 expression in tumor cells and specific immunosuppressive T-cell subsets. **Conclusions:** We developed a potent prognostic model for LUAD and validated SPC25 as a key oncogene and promising therapeutic target.

## Simple Summary

Lung adenocarcinoma outcomes vary greatly because this cancer is highly diverse, making it difficult to predict which patients need more aggressive treatment. To solve this, we combined different types of genetic data to create a new tool based on seven genes. This tool, or “gene signature,” powerfully predicts a patient’s survival chances and reliably identifies high-risk individuals. We discovered that these high-risk tumors are better at hiding from the body’s immune system, which explains why patients with our high-risk score responded poorly to immunotherapy in a real-world study. We then focused on the most important gene in our signature, SPC25. Experiments in cancer cells and animal models confirmed that SPC25 is a key driver of tumor growth; turning it off significantly slowed cancer progression. Our study delivers a practical tool for improving patient prognosis and pinpoints SPC25 as a promising target for the development of new, precise therapies.

## 1. Introduction

Lung cancer remains one of the most prevalent and lethal malignancies worldwide, accounting for the highest cancer-related mortality [1,2,3]. It is categorized into two primary subtypes: non-small cell lung carcinoma (NSCLC), which represents 85% of all cases, and small-cell lung carcinoma (SCLC), constituting the remaining 15% [4]. Lung adenocarcinoma (LUAD) is the predominant histological type of NSCLC [4]. Current standard of care for LUAD includes surgical resection for early-stage disease, platinum-based chemotherapy, and increasingly, targeted therapies against driver mutations (e.g., EGFR, ALK) and immune checkpoint inhibitors [5]. However, the efficacy of these treatments is often hampered by intrinsic or acquired resistance, tumor heterogeneity, and a lack of predictive biomarkers, which ultimately results in a stubbornly low five-year survival rate for LUAD of approximately 20.5% [6]. This stark reality underscores the imperative to discover novel therapeutic targets and construct more reliable prognostic models.

Tumor heterogeneity is a major contributor to this poor outcome, as it drives metastasis, recurrence, and therapy resistance [7]. In LUAD, this heterogeneity manifests predominantly at the genomic, transcriptomic, and proteomic levels, leading to diverse cellular phenotypes and functional variation within the tumor. This complexity underscores the importance of advanced research models, such as patient-derived xenografts, which better preserve tumor heterogeneity and microenvironment characteristics, thereby facilitating more translational research [8]. Additionally, the tumor microenvironment components, particularly cancer-associated fibroblasts and their surface markers, are emerging as crucial players in therapy response and resistance mechanisms [9]. To address these challenges, developing molecular signatures that capture intratumoral heterogeneity is essential for advancing precision medicine. Indeed, previous studies have established numerous prognostic signatures based on gene expression, mutations, or epigenetic alterations, demonstrating their utility in risk stratification and subtype identification [10,11,12,13]. However, many of these models are derived primarily from transcriptomic correlations with patient survival, lacking a direct foundation in functional genetic essentiality data, which may better reflect core tumor vulnerabilities.

In this context, the Dependency Map (DepMap) database provides a powerful resource for identifying genes crucial for tumor survival through genome-scale CRISPR-Cas9 knockout screens. CRISPR-based screens are superior to earlier RNA interference (RNAi) techniques for identifying dependency genes due to their higher efficiency, greater specificity, and reduced off-target effects, enabling more complete gene knockout and thus more reliable identification of essential genes. For instance, CRISPR screens in other malignancies like triple-negative breast cancer have successfully uncovered novel dependencies such as Cop1, which would have been missed by less robust methods [14]. The CERES algorithm quantifies the dependency effect, where lower scores indicate genes more critical for cell fitness [15,16]. These genetic dependencies represent promising therapeutic targets. Integrating such functional genomic data with multi-omics profiles from patient tumors can bridge the gap between in vitro vulnerabilities and in vivo tumor biology, including the complex immune microenvironment. Despite this potential, a comprehensive framework that effectively integrates these dimensions to uncover targetable mechanisms and inform patient-specific treatment in LUAD remains elusive.

**Figure 1:**
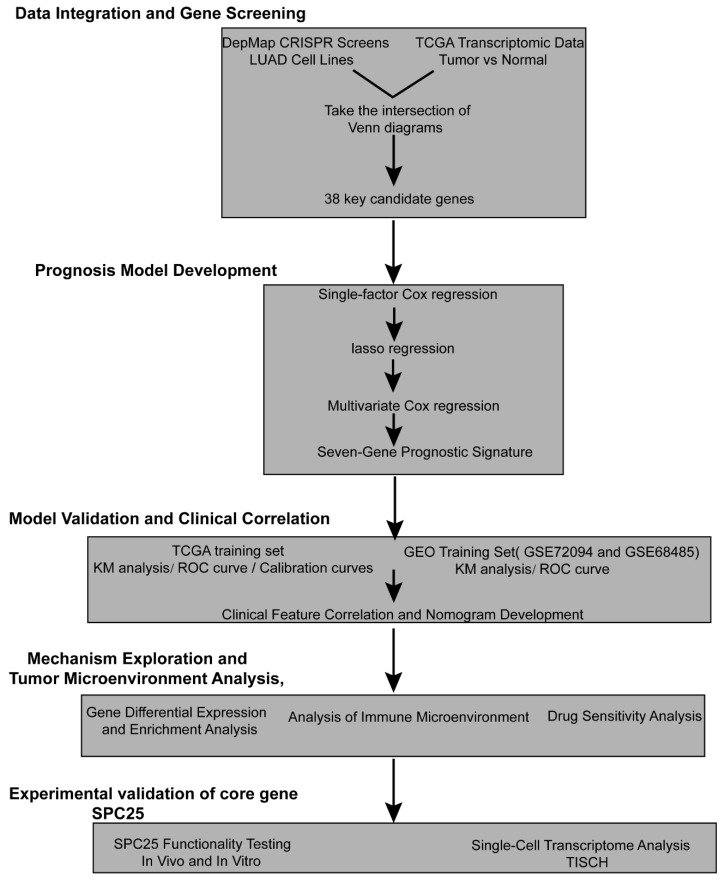
A schematic workflow of the study. The process depicts the key stages, including data acquisition from DepMap and TCGA, prognostic model construction, validation in GEO datasets and an immunotherapy cohort, and experimental validation of the core gene SPC25 in vitro and in vivo.

In the present investigation, we leveraged DepMap data to identify genes critical for LUAD proliferation and survival. By integrating these functional dependencies with multi-omics data from patients, we developed and validated a biologically grounded prognostic signature. This model not only enabled robust risk stratification but also revealed an immunosuppressive microenvironment in high-risk patients and predicted differential responses to immunotherapy, chemotherapy, and targeted agents. To experimentally corroborate the clinical relevance of our model, we validated SPC25—a key gene emerging from our signature—and confirmed its oncogenic role. The primary novelty of our work lies in this integrative framework that prioritizes prognostic genes based on functional essentiality. By demonstrating that a model-derived gene like SPC25 possesses significant biological and clinical relevance, we strengthen the credibility of both the target and the modeling approach itself. This study thus provides a reliable prognostic tool and a validated candidate gene, deepening our comprehension of LUAD heterogeneity and offering a tangible resource to aid clinical decision-making for personalized therapy. The overall design of this integrative approach is summarized in Figure 1. We acknowledge that future validation in more diverse cohorts will be essential to enhance the model’s generalizability and clinical utility.

## 2. Materials and Methods

### 2.1. Data Source

Gene expression data of LUAD were obtained from The Cancer Genome Atlas database (TCGA, https://portal.gdc.cancer.gov/, accessed on 15 May 2024), comprising a total of 600 samples. After excluding samples with tumor recurrence and duplicates, 516 tumor samples and 59 normal samples were retained. Among these, 464 cancer samples with clinical information were used as the training data for the predictive model. Furthermore, data (DepMap Public 22Q2 version) on 1086 tumor cell lines (including 50 lung adenocarcinoma cell lines) subjected to CRISPR-Cas9 gene editing technology were retrieved from the DepMap database (https://depmap.org/portal/, accessed on 15 May 2024). For validation purposes, two LUAD datasets were retrieved from the Gene Expression Omnibus (GEO, https://www.ncbi.nlm.nih.gov/geo/, accessed on 15 May 2024) database: GSE68465 [17], which comprised 462 samples after excluding those with overall survival (OS) less than 30 days and those with missing data, and GSE72094 [18], which included 386 samples following a similar exclusion criterion.

#### 2.1.1. Identification of Crucial LUAD Genes

The study defined lung adenocarcinoma dependence genes (LADGs) if they consistently exhibited CERES scores below −1 across all 50 LUAD cell lines. To discern genes with differential expression between LUAD and healthy lung tissues, the R package ‘DESeq2’ (version 1.40.2) [19] was utilized. DEGs were identified based on an adjusted *p*-value < 0.05 and |log2FC| > 1. Only genes that met both criteria were included as final candidate genes in our analysis.

#### 2.1.2. Construction of a Prognostic Model

A univariate regression analysis was performed on the 38 candidate genes, and only those with a *p*-value less than 0.05 were chosen for subsequent analysis. LASSO regressions were performed using the ‘glmnet’ R package (version 4.1.8) [20], a 10-fold cross-test was performed, and then genes with non-zero λ coefficients were selected for subsequent multivariate Cox regression analyses. The gene coefficients (β) were then estimated using the multivariate Cox regression. Using the β coefficients, risk scores were computed for TCGA patients and stratified by the median score. The same method and threshold were applied to two GEO datasets.

#### 2.1.3. Verification and Assessment of a Predictive Model

The R packages ‘survival’ and ‘survminer’ (version 3.8.3) were employed to generate Kaplan–Meier (KM) survival plots and perform log-rank tests for assessing the discrepancies in survival rates between the high-score risk and low-score risk cohorts. The ‘timeROC’ package (version 0.4) in R was utilized to generate the receiver operating characteristic (ROC) curve and assess the model’s discriminative power through the computation of area under the curve (AUC) metrics at 1, 3, and 5 years. The R package ‘pec’ (version 2023.4.2) [21] was utilized to generate the calibration curve of the model and evaluate the model’s accuracy. Two distinct LUAD datasets sourced from the GEO database were employed as independent verification sets to assess the model’s reliability and accuracy.

### 2.2. Establishment and Validation of a Nomogram Scoring System

Univariate and multivariate Cox regression analyses were performed to assess the independent predictive value of risk scores and clinical parameters on OS. The outcomes were visually represented via a forest plot constructed with the ‘forestplot’ R package (version 3.1.3). Additionally, a predictive nomogram was constructed using R package ‘rms’ (version 6.7.1), which consisted of risk, age, gender and stage. The performance and efficiency of the nomogram were assessed through ROC curves. The accuracy of the nomogram-based prediction model was evaluated using calibration curves. The clinical utility of the nomogram was assessed through decision curve analysis (DCA).

### 2.3. Clinical Analyses Related to Risk Scores

The Wilcoxon rank-sum test was employed to assess disparities in risk scores across various clinical features, including gender, age, and clinicopathological stage, within the TCGA patient cohorts.

### 2.4. Functional Enrichment Analysis Associated with Risk Score

A total of 464 lung adenocarcinoma specimens from the TCGA repository were stratified into high-risk and low-risk categories according to the median risk score. Differential gene expression analysis was performed between the two groups of samples utilizing the ‘Deseq2’ package in R [18]. Volcano plots illustrating the DEGs were created with the R package ‘ggplot2’. DEGs were identified based on an adjusted *p*-value < 0.05 and |log2FC| > 1. The biological roles and signaling cascades linked to the DEGs were explored using Gene Ontology (GO) analysis, Kyoto Encyclopedia of Genes and Genomes (KEGG) analysis and Gene Set Enrichment Analysis (GSEA).

### 2.5. Evaluation of Immune Cell Infiltration

The Estimation of Stromal and Immune Cells in Malignant Tumor Tissues using Expression Data (ESTIMATE) is a widely recognized approach for inferring tumor purity, as well as the levels of immune and stromal cell infiltration within malignant tumor specimens [22]. A quantitative assessment was conducted to compare the ESTIMATE, immune, and stromal scores, as well as tumor purity, between high-risk and low-risk patient groups, employing the ‘estimate’ package (version 1.40.2) in R. In addition, the level of infiltration of immune cell types within the tumor was thoroughly examined by single-sample genomic enrichment analysis (ssGSEA) [23] and the CIBERSORT algorithm [24]. Furthermore, the associations of the risk score with the expression of immune checkpoint molecules and tumor mutation burden (TMB) were evaluated, with TMB data also sourced from the TCGA database. TIDE analysis was used to estimate the effect of immunotherapy in different risk groups [25]. Tumors were classified as “hot” or “cold” using a composite score calculated as: − IDE − MDSC + CD274, based on standardized Z-scores of each parameter. This integrated approach accounts for T-cell dysfunction (TIDE), immunosuppressive cell infiltration (MDSC), and immune checkpoint expression (CD274). Tumors were dichotomized based on the median composite score to define hot (above median) and cold (below median) phenotypes across risk groups.

### 2.6. Chemotherapy Response Prediction

Based on the Genomics of Drug Sensitivity in Cancer (GDSC) database (http://www.cancerrxgene.org/, accessed on 25 Octobor 2024) and the R package ‘oncoPredict’ (version 1.40.2) [26], Chemotherapeutic response prediction was performed for each LUAD sample. The predicted result was a sensitivity score of 198 drugs for each patient. This score correlated positively with the half-maximal inhibitory concentration (IC50). The differences in drug sensitivity (as reflected by the predicted IC50 values) between the high-risk and low-risk groups were statistically compared using the Wilcoxon rank-sum test. A lower IC50 value in a group indicates higher sensitivity to the drug.

### 2.7. Validation of Characteristic Genes

The protein-level expression of the characteristic genes was validated using the Human Protein Atlas (HPA) database (https://www.proteinatlas.org, accessed on 25 Octobor 2024), comparing immunohistochemistry data from tumor tissues with that from paracancerous normal tissues.

### 2.8. Role of Key Genes in Immunotherapy Efficacy and Prognosis for Non-Small Cell Lung Cancer

The Cancer Immunology Data Engine (CIDE, https://cide.ccr.cancer.gov, accessed on 01 Octobor 2025) is an open platform integrating multi-omics data. It incorporates 90 datasets, encompassing 8575 tumor samples from patients treated with immunotherapy across 17 solid tumor types, and can be utilized to systematically identify key genes associated with response to immunotherapy [27]. Based on this platform, we further evaluated a real-world cohort of non-small cell lung cancer (NSCLC) patients (Patil2022-OAK) [28] who received immunotherapy, aiming to investigate the impact of characteristic genes on patient prognosis at different expression levels.

### 2.9. Cell Culture and Lentiviral Transfection

Calu3 and PC9 lung adenocarcinoma cells (Key Laboratory of Respiratory Inflammatory Injury and Precision Diagnosis and Treatment, Chongqing Municipal Health Commission) were cultured in DMEM or RPMI 1640 medium (supplemented with 10% FBS and 1% penicillin/streptomycin), as these are the standard media optimized for the growth of these specific cell lines, respectively. Three distinct SPC25-targeting shRNA sequences and a non-targeting control shRNA were used (Obio Technology, Chongqing, China). Stable SPC25-knockdown lines were established by lentiviral transduction followed by puromycin selection.

### 2.10. Western Blotting (WB)

Total proteins were extracted using RIPA lysis buffer (Beyotime, Chongqing, China), quantified via a bicinchoninic acid (BCA) assay kit (Beyotime), which determines protein concentration through a colorimetric reaction of protein with Cu^2+^ in an alkaline medium. Equal amounts of protein (25 ug per lane) were separated by 12.5% SDS-PAGE and electrophoretically transferred to PVDF membranes using a wet transfer system at 200 mA for 50 min. The membranes were blocked with 5% skimmed milk for 2 h at room temperature and subsequently incubated overnight at 4 °C with primary antibodies: anti-SPC25 (Proteintech, 26474-1-AP) and anti-β-actin (Proteintech, 20536-1-AP). Following TBST washes, the membranes were incubated with a goat anti-rabbit IgG secondary antibody (Abcam, ab6721) for 1 h at room temperature. After additional TBST washes, protein signals were detected using an ECL chemiluminescence kit (MedChemExpress, Chongqing, China).

### 2.11. Colony Formation Assays

Cells (5 × 10^3^/well) were seeded in each well of a 6-well plate for the colony formation experiment. When the colonies were visible to the naked eye (10 Days), the cells were fixed with 4% paraformaldehyde for 15 min and then stained with crystal violet (Beyotime) for 30 min. The clones formed were then counted to reflect the colony forming ability of the clones.

### 2.12. Wound Closure Assays

Cell migration ability was assessed using a wound healing assay. In brief, cells were plated in 6-well plates and cultured to 80–100% confluence in complete growth medium. A uniform scratch wound was created using a sterile 200 μL pipette tip. The dislodged cells were removed by washing twice with PBS, and then fresh serum-free medium was added to minimize the influence of cell proliferation. The plates were incubated under standard culture conditions (37 °C, 5% CO_2_). Microscopic images of identical fields were captured at 0 and 48 h using an inverted microscope (Olympus IX73, Japan) at 100× magnification. The wound closure percentage was quantified using ImageJ software (Version 1.54f) by measuring the residual cell-free area at 48 h relative to the initial wound area at 0 h, as previously described [29].

### 2.13. Ethynyl Deoxyuridine (EDU) Proliferation Assay

Cells (2 × 10^5^/well) were seeded in 24-well plates. After attachment, cultures were pulsed with 10 μM EdU labeling medium (Beyotime) for 6 h (37 °C, 5% CO_2_). Cells were fixed with 4% paraformaldehyde (15 min), followed by Click-iT reaction to detect EdU according to the manufacturer’s protocol. Nuclei were counterstained with DAPI, and images were acquired using an inverted fluorescence microscope (Olympus IX73, Japan).

### 2.14. Animal Experiments

Male BALB/c nude mice (4 weeks old) were obtained from Vital River (China) and housed under SPF conditions for one week prior to experimentation. All procedures were approved by the IACUC of Chongqing medical university (Protocol No. IACUC-SAHCQMU-2025-0185). For the subcutaneous xenograft model, Calu3 cells stably transfected with sh-NC or sh-SPC25#3 (1 × 10^7^ cells per mouse) were resuspended in 100 μL of PBS and inoculated into the right flank of each mice (*n* = 3 per group). Tumor size was measured every other day with a digital caliper, and the volume was calculated as 1/2 × (length × width^2^). When the tumors in the control group reached a volume of about 1000 mm^3^ (at day 28 post-inoculation), all mice were euthanized, and the tumors were harvested and weighed.

### 2.15. Single Cell Sequencing Analysis

The single-cell RNA sequencing data from the NSCLC_EMTAB6149 dataset [30] was subjected to a rigorous screening process on the TISCH [31] platform (http://tisch.comp-genomics.org/home/, accessed on 15 May 2024) to identify and extract key information fragments.

### 2.16. Statistical Analysis

Data processing and statistical analyses were performed using R software (version 4.3.1). All experiments were conducted with at least three biological replicates. Student’s *t*-test was applied when both groups exhibited normal distributions with confirmed homogeneity of variance. For comparisons between two groups violating normality assumptions, the Wilcoxon rank-sum test was employed. Statistical significance was defined as *p* < 0.05 and denoted as follows: ns (*p* > 0.05), * (*p* < 0.05), ** (*p* < 0.01), *** (*p* < 0.001), **** (*p* < 0.0001).

## 3. Results

### 3.1. Identifying Lung Adenocarcinoma Dependence Genes (LADGs) and Developing Prognostic Signature

**Figure 2:**
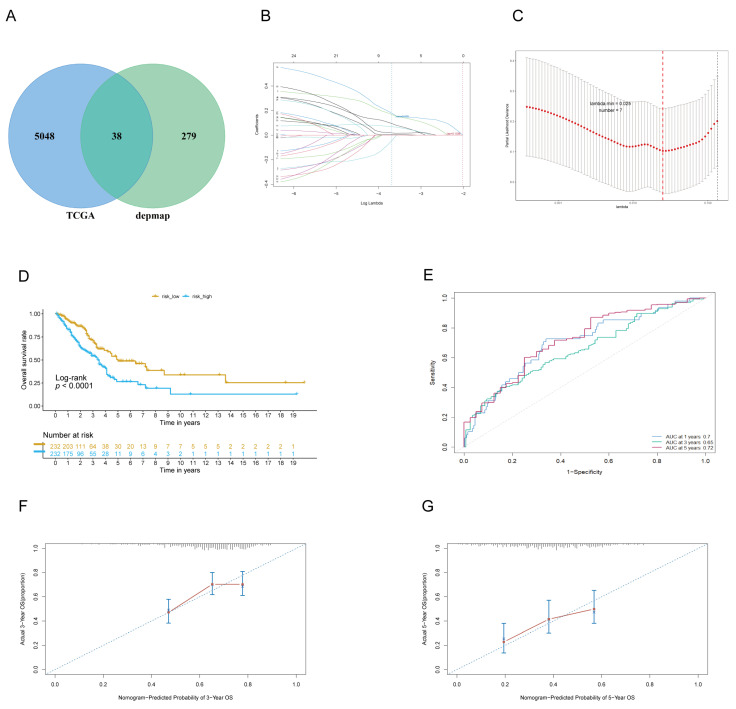
The TCGA cohort established a prognostic risk score model. (**A**): Venn Diagram obtained by intersecting TCGA and Depmap data. (**B**,**C**): Analysis of LASSO coefficient profiles coupled with cross-validation to pinpoint the most influential prognostic genes. (**D**): KM curves for the TCGA cohort based on different risk scores. (**E**): Time-dependent ROC curves at one year, three years, and five years in the TCGA dataset. (**F**,**G**): Calibration curves for the TCGA cohort prediction model at 3 years (**F**) and 5 years (**G**) were constructed separately.

In this study, we identified 317 LADGs from the DepMap database and 5086 DEGs from the TCGA data. Among these, 38 genes were found to be common between the two gene sets (Figure 2A). Furthermore, we performed univariate regression analyses on these 38 genes and identified 32 genes with prognostic value. Subsequently, 7 genes with nonzero coefficients were identified through LASSO regression analysis and used to build a multivariate Cox regression prognostic model (Figure 2B,C). This prognostic model signature’s risk score was determined using the following formula: Risk score = (0.302554867 × expr (CCT6A)) + (−0.421506186 × expr (MCM7)) + (0.220146569 × expr (HSPE1)) + (0.103271215 × expr (H2BC4)) + (−0.007844594 × expr (RRM2)) + (0.387533403 × expr (PLK1)) + (0.047518089 × expr (SPC25)).

Based on the median risk score, patients in the TCGA cohort were categorized into high-score (*n* = 232) and low-score risk cohorts (*n* = 232). KM survival curves showed that OS was significantly lower in the high-risk group than in the low-risk group (Figure 2D). Subgroup analyses further demonstrated that this prognostic stratification was particularly effective in early-stage (Stage I–II) patients (Log-rank *p* < 0.001), highlighting its potential clinical utility for this subgroup (Supplementary Material S1). The performance of the LADGs feature was assessed employing a temporal ROC curve, indicating good predictive capability and effectiveness in forecasting OS. The AUC values for 1-, 3-, and 5-year survival were 0.70, 0.65, and 0.72, sequentially (Figure 2E). These robust AUC values validate the competitive performance of our model against existing prognostic signatures for LUAD (Supplementary Material S2). Moreover, the validity of the LADGs features was confirmed by calibration plots, which demonstrated a strong agreement between the observed outcomes and the predicted probability of survival for both 3-year and 5-year survival rates (Figure 2F,G). This finding underscores the robustness of our LADGs in forecasting unfavorable outcomes among patients with lung adenocarcinoma.

**Figure 3:**
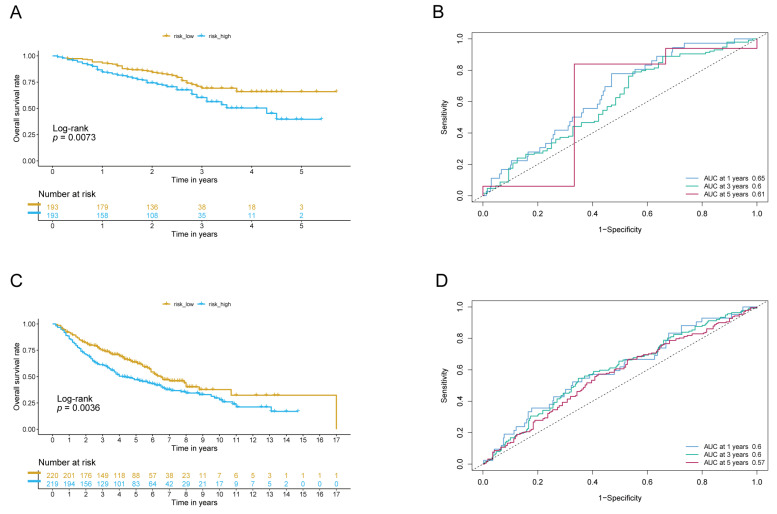
The predictive capacity of the risk score models was evaluated using two independent datasets. (**A**,**B**): The KM survival curve (**A**) and the ROC curve (**B**) for the first year, third year, and fifth year were generated for the GSE72094 dataset. (**C**,**D**): The KM survival curve (**C**) and the ROC curve (**D**) for the first year, third year, and fifth year were generated for the GSE68465 dataset.

The LADGs signature was validated in two distinct GEO datasets, yielding results that were in line with those from the TCGA sample. The clinical and pathological attributes of the patient samples are summarized in Table 1. Notably, the high-score risk cohorts demonstrated significantly higher mortality rates and inferior overall survival compared to the low-score risk cohorts in both validation cohorts, GSE72094 (Figure 3A) and GSE68485 (Figure 3C). A ROC curve for both validation cohorts indicated that an average AUC values for 1-, 3-, and 5-year survival were capable of reaching 0.6 (Figure 3B,D). In conclusion, the LADGs signature demonstrated its efficacy as a dependable prognostic indicator for LUAD patients.

### 3.2. The Signature of LADGs Was Identified as a Significant Independent Prognostic Factor for LUAD

**Figure 4:**
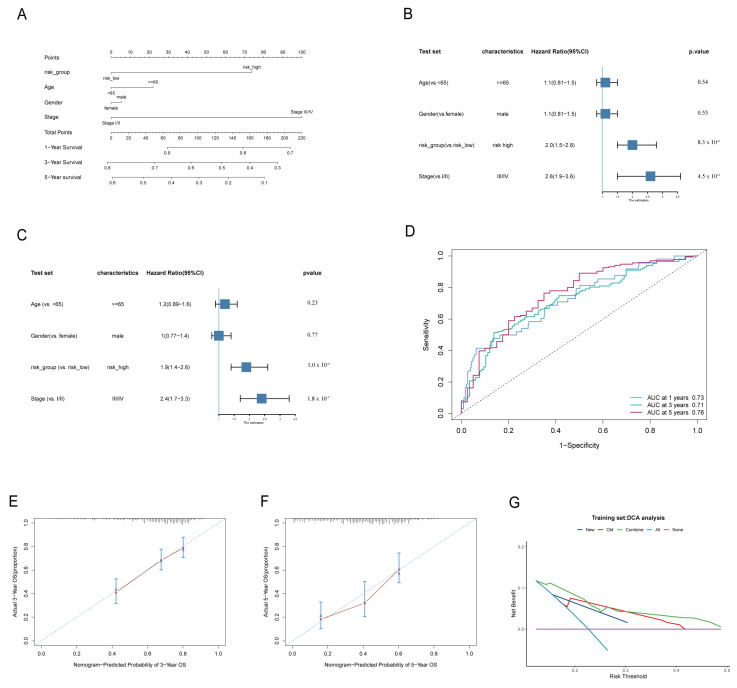
The TCGA cohort constructed a risk score-based nomogram. (**A**): Nomogram model for prediction of 1-year, 3-year and 5-year OS in the TCGA cohort. (**B**,**C**): The univariate (**B**) and multivariate (**C**) Cox regression analyses for TCGA cohorts are intuitively represented by forest maps. (**D**): Time-dependent ROC curves at one year, three years, and five years. (**E**,**F**): Calibration curves for the nomogram at 3 years (**E**) and 5 years (**F**) were constructed separately. (**G**) Decision curve analysis (DCA) for the nomogram and individual risk factors.

The investigation meticulously examined clinical attributes, encompassing age, sex, risk stratification, and tumor staging, to devise a prognostic nomogram (Figure 4A) with the objective of forecasting the 1-year, 3-year, and 5-year survival probabilities for individuals with LUAD. Findings from Cox proportional hazards regression analyses explored that a risk stratification (univariate Cox regression: hazard ratio [HR] = 2.0, 95% confidence interval [CI] 1.5–2.8; multivariate Cox regression: HR = 1.9, 95% CI 1.4–2.6) was a significant independent prognostic indicator for LUAD patients (Figure 4B,C). The nomogram’s efficacy and predictive power were confirmed and quantified using ROC curve analysis within the TCGA training cohort. The AUC values were 0.73, 0.71, and 0.76 for the 1-year, 3-year, and 5-year survival rates, respectively (Figure 4D). Additionally, calibration plots were explored to evaluate the predictive accuracy of the model derived from the nomogram. The calibration plots for the 3-year and 5-year survival rates aligned closely with the 45-degree reference line, suggesting a strong concordance between the predicted and actual survival rates within the TCGA training set (Figure 4E,F). DCA demonstrated that the nomogram conferred greater clinical utility compared to any individual risk factor (Figure 4G).

### 3.3. The Correlation Between Signature of LADGs and Clinical Characteristics

**Figure 5:**
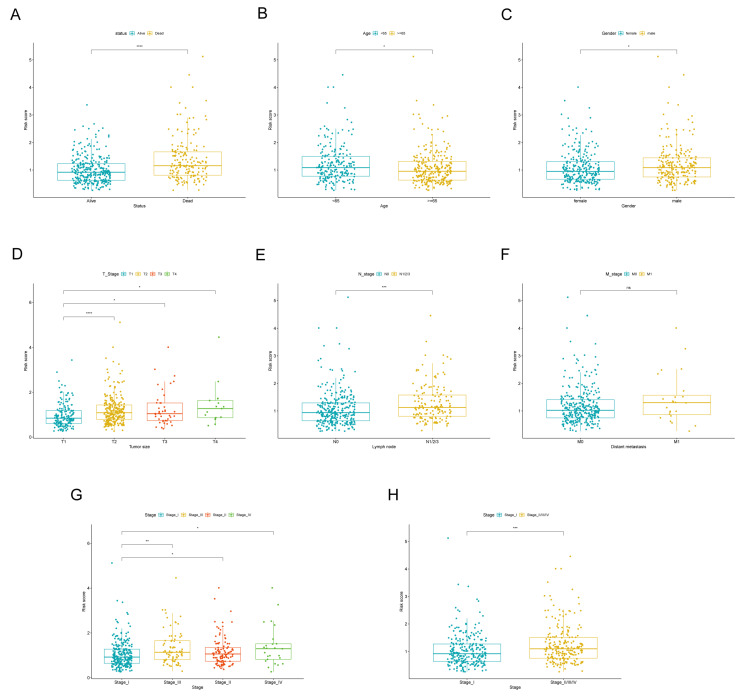
Difference in risk scores among subgroups based on clinical characteristics. The Wilcoxon rank-sum test was employed to assess the disparities in risk scores across various patient status (**A**), age (**B**), gender (**C**), tumor size (**D**), lymph node involvement (**E**), distant metastasis (**F**), and pathological stage categories (**G**,**H**) within the TCGA cohort.

A comparison was conducted between the risk of patients with different clinical status (death vs. survival), age (<65 vs. ≥ 65), gender (female vs. male), tumor size (T1 vs. T2 vs. T3 vs. T4), lymph nodes (N0 vs. N1-N3), distant metastasis (M0 vs. M1), and pathological stages (stage I vs. stage II to IV) in the TCGA cohort. Patients who died had significantly higher risk scores than those who survived (Figure 5A). Patients older than 65 years had a lower score than patients younger than 65 years (Figure 5B). Males exhibited a higher risk stratification compared to females (Figure 5C). Furthermore, patients with T2, T3, and T4 tumor stages demonstrated elevated risk scores in contrast to those with T1 tumors (Figure 5D). The risk score for N1-N3 lymph node involvement was found to be greater than that for N0 (Figure 5E). Notably, the risk score for stage II-IV disease was significantly higher than for stage I (Figure 5G). All these findings were statistically significant. While the risk score for M1 distant metastasis was higher than for M0, this difference was not statistical significance, which we speculate could be attributed to the limited number of M1 patients within the TCGA-LUAD dataset. In our analysis comparing risk scores with distant metastasis, we excluded patients with an unknown metastatic status (Mx stage) (Figure 5F). In sum, we found that higher risk scores were associated with higher pathological stage and poor prognosis.

### 3.4. Differential Gene Analysis and Enrichment Analysis in High-Score and Low-Score Risk Groups on the LADGs Signature

**Figure 6:**
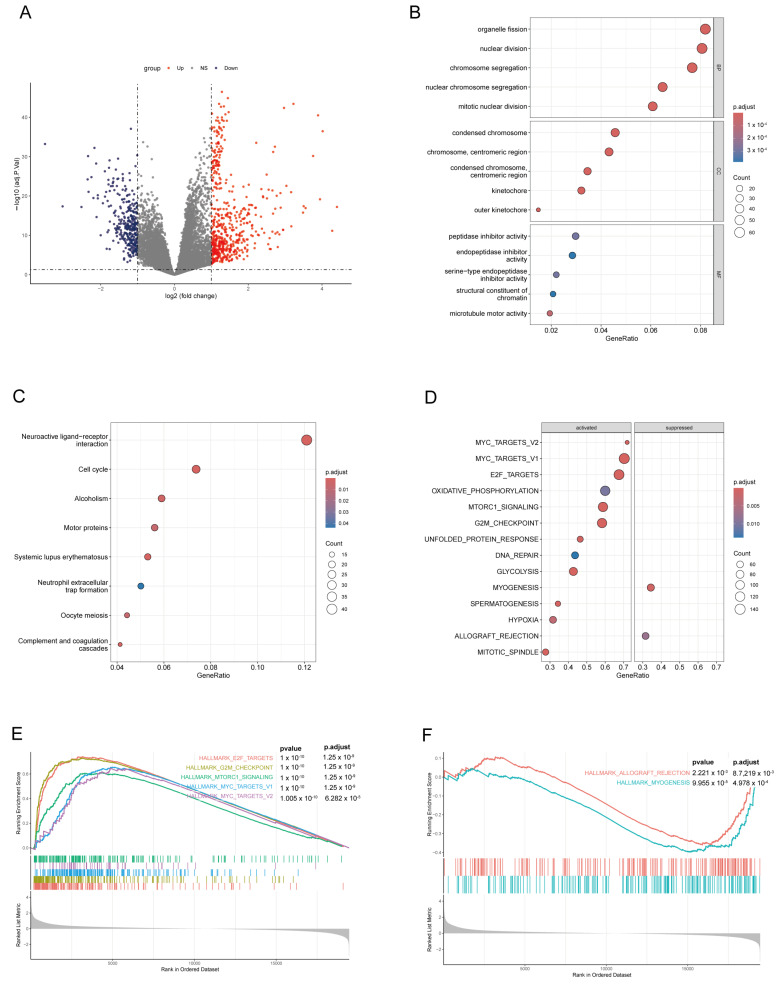
Differentially expressed genes were analyzed in patients with high and low risk scores in the TCGA cohort. (**A**): A volcano plot of the differentially expressed genes. (**B**–**D**): GO (**B**), KEGG (**C**) and GSEA (**D**) analysis results for differentially expressed genes. (**E**,**F**) The GSEA identified a series of pathways that were either significantly activated (**E**) or inhibited (**F**).

The Deseq2 package was employed for the assessment of variance in gene expression between high-risk and low-risk patients. This analysis revealed 878 genes exhibiting significant differences (adjusted *p*-value < 0.05 and absolute value of log fold change >1), comprising 553 genes showing up-regulation and 325 genes showing down-regulation (Figure 6A). Subsequently, GO and KEGG analyses were conducted on the differentially expressed genes. A GO analysis showed that the DEGs were enriched in cell division, metabolic regulation and immune response pathways (Figure 6B). The KEGG analysis explored that several pathways, including Neuroactive ligand-receptor interaction, Cell cycle, Neutrophil extracellular trap formation, and complement and coagulation cascades, could be linked to a progression and onset of LUAD (Figure 6C). These pathways involve components that are pertinent to intracellular signaling, DNA damage and repair, inflammation, and angiogenesis.

A TCGA-LUAD cohort was analyzed using GSEA to investigate the enrichment of the HALLMARKS gene set. The outcomes were illustrated via a bubble diagram, with the key pathways selected and presented separately (Figure 6D). The two suppressed pathways were Hallmark myogenesis, which includes the coordination of different signaling pathways, transcription factors, and structural proteins, and Hallmark allograft rejection, which involves both innate and adaptive immune responses (Figure 6E). The most activated 6 pathways were Hallmark E2F targets, Hallmark MYC targets V1, Hallmark MYC targets V2, Hallmark Oxidative phosphorylation, Hallmark mTORC1 signaling, and Hallmark G2M checkpoint (Figure 6F). Hallmark E2F targets and the Hallmark G2M checkpoint play crucial roles in regulating the cell cycle, and it has been demonstrated that abnormalities in the cell cycle are closely associated with tumor development. Hallmark MYC targets V1 and Hallmark MYC targets V2 can enhance cell proliferation and suppress apoptosis by modulating the transcription factor MYC, which is frequently overexpressed in tumor cells. Hallmark Oxidative phosphorylation and Hallmark mTORC1 signaling impacts tumor growth and invasion by modulating the energy metabolism characteristics of tumor cells. To summarize, the investigation revealed substantial alterations in pathways linked to cell growth, programmed cell death, metabolic processes, and immune response among patients categorized as high- and low-risk.

### 3.5. The Link Between LADGs Signature and Tumor Microenvironment

**Figure 7:**
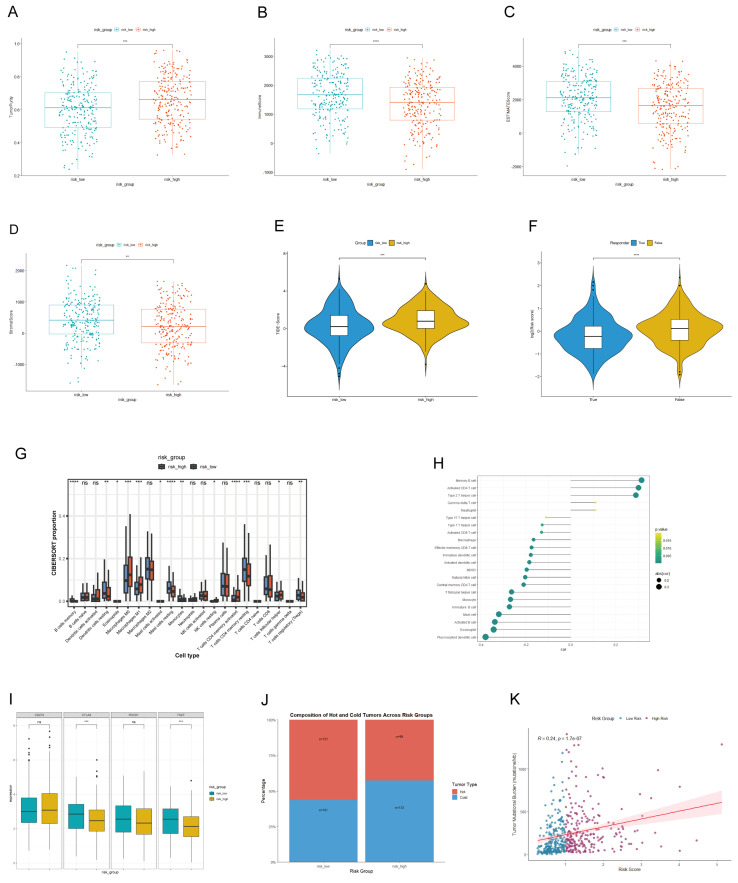
Tumor microenvironment and immunogenomic profiling based on risk score in the TCGA-LUAD cohort. Comparison of (**A**) tumor purity, (**B**) immune score, (**C**) ESTIMATE score, and (**D**) stromal score between the high-risk and low-risk groups. (**E**) Immune cell infiltration levels assessed by ssGSEA. (**F**) Proportional abundances of immune cell types evaluated by CIBERSORT. (**G**) Comparison of TIDE scores between the high- and low-risk groups. (**H**) Analysis of immunotherapy responses. (**I**) Expression differences in key immune checkpoint molecules between the two risk groups. (**J**) The distribution of “hot” and “cold” tumors across risk groups. (**K**) Correlation between the risk score and tumor mutation burden (TMB).

We comprehensively characterized the tumor microenvironment (TME) associated with the risk signature in LUAD. The high-risk group demonstrated significantly elevated tumor purity but reduced stromal, immune, and ESTIMATE scores (Figure 7A–D). Immune infiltration analyses revealed an enrichment of immunosuppressive cells (e.g., M0 macrophages) and a depletion of effector populations (e.g., memory B cells, M2 macrophages) in this group (Figure 7G,H). Supporting this immunosuppressive phenotype, the high-risk group showed higher TIDE scores and poorer predicted response to immunotherapy (Figure 7E,F). Notably, although risk score correlated positively with tumor mutation burden (TMB; Figure 7K), the high-risk group was predominantly “cold” tumors, whereas low-risk tumors were enriched for “hot” phenotypes (Figure 7J). This divergence was further reflected in immune checkpoint expression: CTLA-4 and TIGIT were significantly upregulated in the low-risk group, consistent with a T-cell-inflamed TME, while PD-1/PD-L1 showed no intergroup difference (Figure 7I). Collectively, these results indicate that the strongly immunosuppressive TME in high-risk patients overrides any potential benefit from high TMB, explaining their diminished response to immunotherapy.

### 3.6. Prediction of Drug Treatment Response Based on LADGs Features

**Figure 8:**
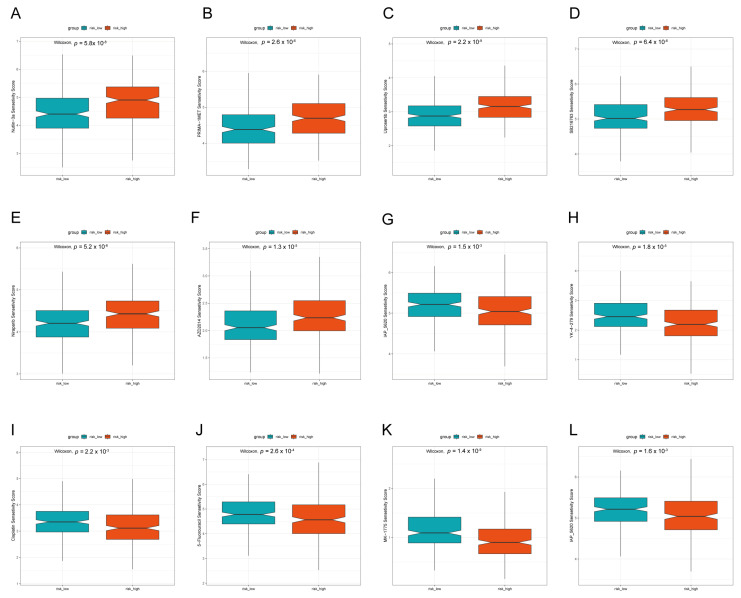
Assessment of drug sensitivity in the TCGA-LUAD cohort in the high- and low-risk groups. Drugs with lower IC50 and *p* < 0.05 were selected for presentation in the high- (**A**–**F**) and low-risk groups (**G**–**L**). (**A**) Nutlin-3a, (**B**) PRIMA-1MET, (**C**) Uprosertib, (**D**) SB216763, (**E**) Niraparib, (**F**) AZD2014, (**G**) Lapatinib, (**H**) YK-4-279, (**I**) Cisplatin, (**J**) 5-Fluorouracil, (**K**) MK-1775, (**L**) IAP_5620.

The drug sensitivity was examined in two risk groups based on the sensitivity scores generated by the R package ‘oncoPredict’ (Figure 8). The findings indicated that the low-risk cohort demonstrated reduced IC50 values across various therapeutic agents, such as Nutlin-3a (an MDM2 inhibitor), PRIMA-1MET (a p53 activator), Uprosertib (an AKT signaling pathway inhibitor), SB216763 (a GSK-3 inhibitor), Niraparib (a PARP inhibitor), and AZD2014 (an mTOR inhibitor). Conversely, the high-risk cohort exhibited lower IC50 scores for certain drugs, such as Lapatinib (HER2 and EGFR inhibitors), YK-4-279 (EWS-FLI1 inhibitor), Cisplatin (cell cycle inhibitor), 5-Fluorouracil (Thymine nucleotide synthetase inhibitor), MK-1775 (WEE1 inhibitor), and IAP_5620 (IAP inhibitor). The predicted sensitivity of the low-risk group to p53-targeting agents (Nutlin-3a and PRIMA-1MET) is mechanistically supported. As Nutlin-3a activates wild-type p53 and PRIMA-1MET reactivates mutant p53 [32], the efficacy in our low-risk group implies these tumors rely on functional p53 pathways—a finding consistent with prior studies on PRIMA-1MET context-specific efficacy. In conclusion, the prediction of which chemotherapeutic agents should be prescribed based on tumor heterogeneity in different risk groups facilitates the provision of more personalized treatment for patients.

### 3.7. Expression and Localization of Signature Genes

**Figure 9:**
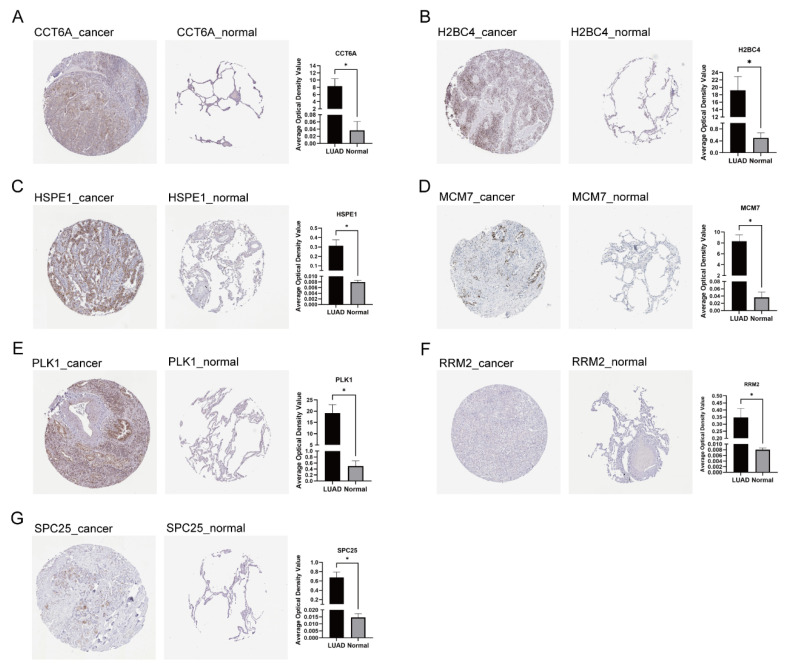
Immunohistochemical results of LADGs in LUAD and normal lung tissues according to the Human Protein Atlas (HPA) database. (**A**) CCT6A. (**B**) H2BC4. (**C**) HSPE1. (**D**) MCM7. (**E**) PLK1. (**F**) RRM2. (**G**) SPC25.

The expression levels and cellular localization of the signature genes were validated using immunohistochemical staining data from lung adenocarcinoma and normal tissue specimens obtained from the Human Protein Atlas database (HPA, https://www.proteinatlas.org/, accessed on 25 Octobor 2024). Specifically, CCT6A, HSPE1, RRM2, and SPC25 were localized in the cytoplasm, whereas H2BC4, MCM7, and PLK1 were localized in the nucleus. Notably, the seven genes expression levels were differentially elevated in tumor tissues compared to normal tissues (Figure 9).

### 3.8. Characteristic Genes Hold Promise as Biomarkers and Potential Targets for Immunotherapy

**Figure 10:**
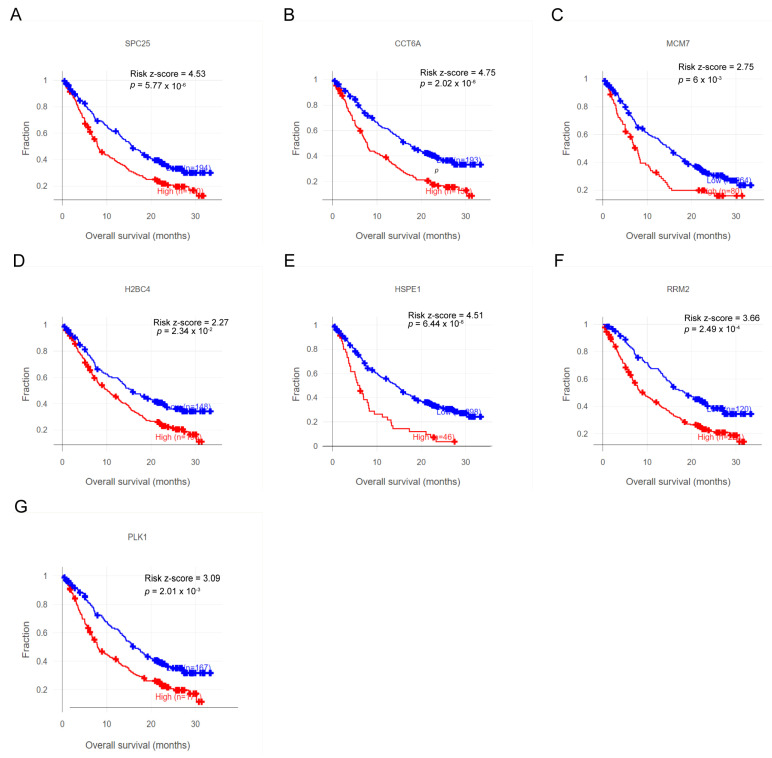
High expression of characteristic genes is associated with poor overall survival in NSCLC patients receiving immunotherapy. (**A**) SPC25. (**B**) CCT6A. (**C**) MCM7. (**D**) H2BC4. (**E**) HSPE1. (**F**) RRM2. (**G**) PLK1.

Our analysis of the real-world NSCLC cohort (Patil2022-OAK) via the CIDE platform revealed a significant association between high expression of all prognostic characteristic genes and reduced overall survival (OS) post-immunotherapy (Figure 10A–G). Notably, this association was statistically significant for each individual gene. Collectively, these results indicate the strong potential of these genes as a prognostic biomarker and a basis for developing novel therapeutic strategies in immunotherapy for NSCLC.

### 3.9. Verification of LADGs Expression and Function in LUAD Cells

**Figure 11:**
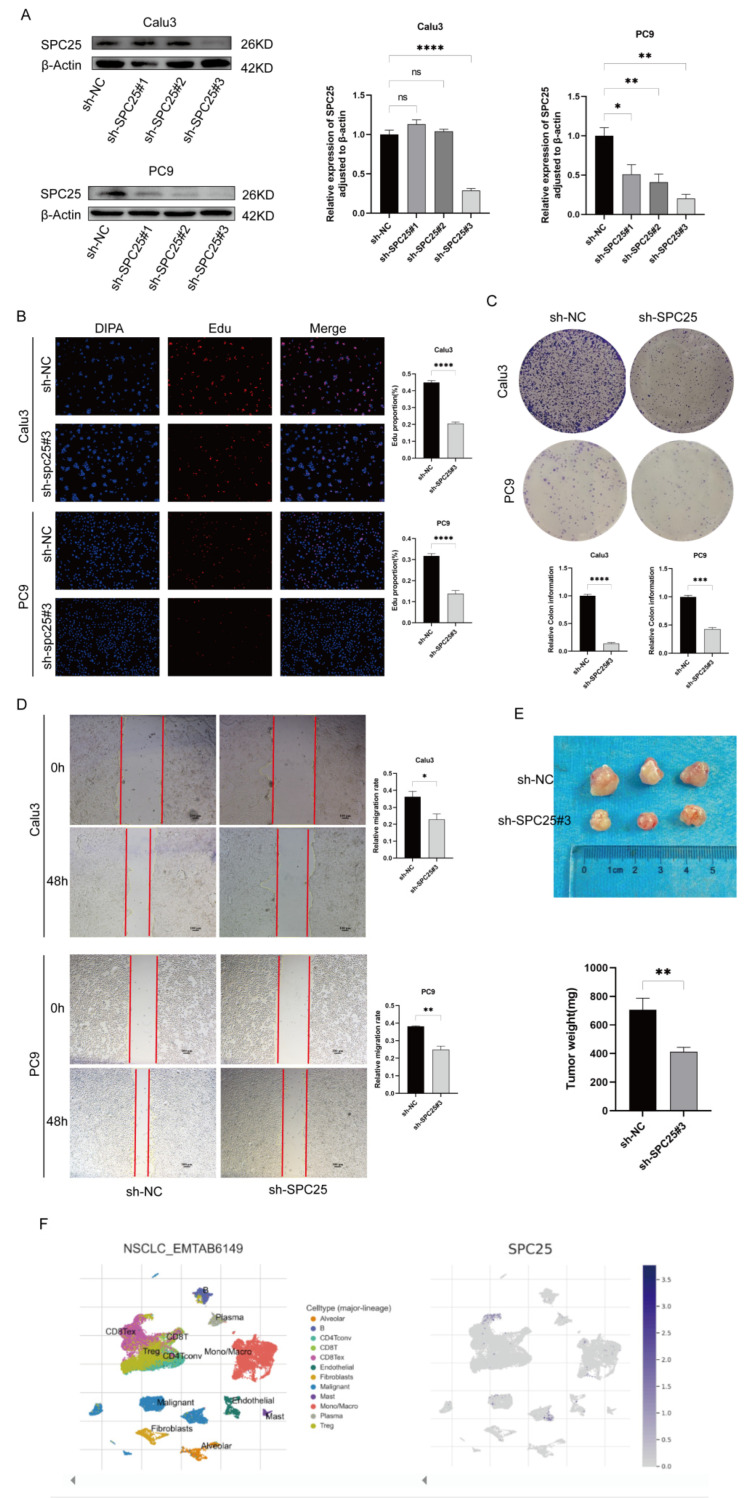
Functional characterization of SPC25 in LUAD. (**A**) Western Blot analysis confirmed SPC25 knockdown efficiency in Calu3 and PC9 cells. (**B**) EdU assays were performed to measure cell proliferation in control cells versus SPC25-knockdown cells. (**C**) Colony formation assays validated the clonogenic ability of Calu3 and PC9 cells following SPC25 knockdown. (**D**) Wound healing assays demonstrated differences in migration capacity between control and SPC25-knockdown cells. (**E**) In Vivo tumor formation assay. Representative images of xenograft tumors derived from control and SPC25-knockdown cells and the corresponding tumor weight statistics are shown. (**F**) Single-cell sequencing analysis.

To validate the role of a key gene from our prognostic model, we knocked down SPC25 in LUAD cells (Calu3 and PC9). Western blot confirmed efficient knockdown (Figure 11A; see Supplementary Material S3 for full membranes). Functional assays demonstrated that SPC25 depletion significantly impaired tumor cell proliferation (EdU assay, Figure 11B), clonogenicity (colony formation assay, Figure 11C), and migration (wound healing assay, Figure 11D). The critical oncogenic role of SPC25 was further confirmed in vivo; in a subcutaneous xenograft model, tumors derived from SPC25-knockdown Calu3 cells exhibited significantly slower growth and lower final weight compared to the control group (Figure 11E). Single-cell sequencing analysis revealed that SPC25 is predominantly highly expressed in tumor cells and specific immunosuppressive cell populations, such as CD8-depleted T cells and Tregs (Figure 11F). Collectively, these findings from cellular, animal, and clinical data establish SPC25 as a critical promoter of LUAD malignancy, linking its pro-tumorigenic functions to an immunosuppressive microenvironment, and nominating it as a promising therapeutic target.

## 4. Discussion

Lung cancer ranks among the most common types of cancer globally, posing a serious threat to both quality of life and health [2]. Among its various subtypes, lung adenocarcinoma (LUAD) is the most common, and improving the prognosis of patients with this subtype has garnered significant attention. However, the traditional TNM pathological staging system fails to adequately account for certain characteristics of lung cancer patients, including those with LUAD. Despite sharing the same pathological type and tumor stage, or receiving similar treatment, there exist notable variations in the prognosis of cancer patients. While some individuals successfully combat the disease, others may encounter tumor recurrence or progression. The construction of a prognostic model based on multi-gene features related to specific biological processes can assist in the identification and description of tumor heterogeneity, as well as in the development of personalized treatment plans.

Dependency genes are those that are essential for the development and survival of cancer cells [33]. They confer properties that distinguish cancer cells from normal cells, thereby providing new insights into our understanding of tumor development, identification of tumor vulnerability, and development of personalized therapeutic strategies [34]. The Dependency Map (DepMap) database integrates a CRISPR-Cas9 screening dataset of 1186 cancer cell lines and applies the CERES scoring system [15,16]. In this study, we identified a group of genes, named Lung Adenocarcinoma Dependence Genes (LADGs), that are essential for LUAD cell proliferation and growth, using CERES scores from the DepMap project. We then constructed a 7-gene prognostic risk model based on these LADGs and assessed its accuracy. The results demonstrated that the risk scores based on the characteristics of LADGs can serve as a significant independent prognostic factor and are significantly correlated with higher pathological stage and poor prognosis. To elucidate the prognostic discrepancies between high-score and low-score risk patients, we performed differential gene expression, functional enrichment, immune infiltration, and drug sensitivity analyses. Finally, for clinical applications, combining age, gender, risk score group and cancer stage, we constructed a nomogram and validated its predictive efficiency and accuracy.

The seven-gene prognostic signature comprises CCT6A, MCM7, HSPE1, H2BC4, RRM2, PLK1, and SPC25. CCT6A promotes metastasis in liver cancer via metabolic reprogramming mediated by RPS3 [35], drives LUAD progression through the STAT1/HK2/glucose metabolism axis [36], enhances stemness in oral cancer via activation of the Wnt/Notch pathway [37], and activates the PI3K/AKT signaling pathway to promote epithelial–mesenchymal transition (EMT) and proliferation in breast cancer [38]. MCM7 enhances stemness via autophagy in bladder cancer [39] and is associated with cisplatin resistance in both bladder cancer [40] and liver cancer via PI3K/AKT signaling [41]. HSPE1 modulates ferroptosis by regulating GPX4/lipid peroxidation in bladder cancer [42] and promotes LUAD malignancy via aerobic glycolysis [43]. H2BC4 is a prognostic biomarker in pancreatic cancer [44] and is associated with gemcitabine resistance in LUAD [45]. RRM2 stabilizes ANXA1/activates AKT to confer resistance to sunitinib and PD-1 blockade in renal cancer [46], is linked to docetaxel resistance in prostate cancer [47], activates TGF-β to drive pancreatic cancer progression/metastasis [48], and in Head and Neck Squamous Cell Carcinoma (HNSCC), binds TXNRD1 to regulate PD-L1/redox balance; targeting TXNRD1 enhances immunotherapy-mediated ferroptosis via increased CD8+ T cells and reduced PD-L1 [49]. PLK1 is pivotal, controlling transcription factors to promote proliferation and EMT [50]; its inhibition upregulates PD-L1, stimulating immunity and sensitizing pancreatic cancer to immunotherapy [51], mediates palbociclib resistance in metastatic breast cancer [52], and PLK1 inhibitors combined with abiraterone suppress tumor growth in prostate cancer [53]. SPC25 is associated with cisplatin resistance and stemness in HNSCC [54], promotes stemness and predicts survival in LUAD [55], mediates immune escape via glutamine metabolism in LUAD [56], and its inhibition overcomes stemness and enhances platinum sensitivity in ovarian cancer [57]. Notably, in the independent Patil2022-OAK cohort, high expression of every gene in our signature independently predicted worse survival after immunotherapy, affirming their collective utility as a prognostic biomarker and highlighting their potential as therapeutic targets.

Our analysis revealed that the aggressive phenotype of the high-risk group is associated with an immunosuppressive TME [58], characterized by elevated tumor purity, diminished immune infiltration, and an enrichment of immunosuppressive cells. This “cold” tumor phenotype is further corroborated by a higher TIDE score, predicting a diminished response to immunotherapy. Conversely, the low-risk group’s “hot” TME suggests a pre-existing immune response. This dichotomy extends to differential drug sensitivities, offering a roadmap for personalized therapy. The low-risk group showed heightened sensitivity to agents targeting p53 and specific signaling pathways, while high-risk tumors were more vulnerable to classic chemotherapeutics and DNA damage repair inhibitors. The clinical relevance of our signature is strongly supported by functional validation. We demonstrated that SPC25, a key component of our model, is a functional oncogene. Its knockdown significantly impaired tumor cell proliferation, clonogenicity, and migration in vitro and curtailed tumor growth in vivo. Single-cell sequencing analysis positioned SPC25 within both tumor cells and immunosuppressive T-cell populations, mechanistically linking its pro-tumorigenic role to the establishment of an immunosuppressive microenvironment.

In summary, our study extends beyond the construction of a prognostic model by providing multi-faceted insights into the immunosuppressive TME, distinct therapeutic vulnerabilities, and functional oncogenic mechanisms associated with the LADG signature.

## 5. Conclusions

In conclusion, this study successfully developed and validated a novel 7-gene prognostic signature based on lung adenocarcinoma dependence genes (LADGs). The signature demonstrated robust performance in predicting overall survival and was established as an independent prognostic factor for LUAD patients. Through comprehensive bioinformatics analyses and experimental validation, we revealed the critical roles of these LADGs in tumor proliferation, metastasis, metabolic reprogramming, and immune modulation. The signature’s significant association with the tumor microenvironment and drug sensitivity profiles provides valuable insights for developing personalized treatment strategies, particularly in immunotherapy.

Notwithstanding these findings, several limitations of our study should be acknowledged. Firstly, the primary analysis was conducted using publicly available databases, which may contain inherent biases and lack certain clinical details. Future prospective, multicenter studies with substantial real-world sample sizes are necessary for clinical validation. Secondly, while we validated the oncogenic role of SPC25 through in vitro and in vivo experiments, the functional characterization of the other six genes in our signature remains to be fully elucidated. Thirdly, the practical application of our risk score model requires certain technical expertise and resource inputs, which might limit its immediate widespread clinical adoption. Despite these limitations, our study provides a valuable framework for understanding genotype-specific vulnerabilities in LUAD and offers expanded.

## Figures and Tables

**Table 1:** The clinicopathological characteristics of the datasets.

Characters	Level	TCGA	GSE68465	GSE72094
N		464	439	386
Gender	Male	212	221	168
	Female	252	218	218
Age (year)	<65	207	213	104
	>=65	257	226	282
T stage	T1	159	150	NA
	T2	243	248	NA
	T3	42	29	NA
	T4	17	11	NA
	Tx	3	NA	NA
N stage	N0	301	287	NA
	N1	84	87	NA
	N2	67	52	NA
	N3	2	NA	NA
	Nx	10	1	NA
M stage	M0	303	NA	NA
	M1	24	NA	NA
	Mx	134	NA	NA
	Missing	3	NA	NA
Pathologic stage	I	253	NA	246
	II	108	NA	65
	III	78	NA	56
	IV	25	NA	14
	Missing	NA	NA	5
Race	White	362	291	365
	Non-white	58	19	18
	Missing	44	129	3
Smoker	Yes	NA	297	291
	No	NA	49	65
	Missing	NA	93	30
Histological type	Acinar cell carcinoma	20	NA	NA
	Adenocarcinoma with mixed subtypes	103	NA	NA
	Adenocarcinoma, NOS	280	NA	NA
	Bronchio-alveolar carcinoma, mucinous	4	NA	NA
	Bronchiolo-alveolar adenocarcinoma, NOS	3	NA	NA
	Bronchiolo-alveolar carcinoma, non-mucinous	14	NA	NA
	Clear cell adenocarcinoma, NOS	1	NA	NA
	Micropapillary carcinoma, NOS	2	NA	NA
	Mucinous adenocarcinoma	11	NA	NA
	Papillary adenocarcinoma, NOS	20	NA	NA
	Signet ring cell carcinoma	1	NA	NA
	Solid carcinoma, NOS	5	NA	NA
OS status	Alive	297	206	277
	Dead	167	233	109
Treatment type	Pharmaceutical Therapy, NOS	233	89	NA
	Radiation Therapy, NOS	231	65	NA

## Data Availability

The original contributions presented in the study are included in the article/Supplementary Material. Further inquiries can be directed to the corresponding authors.

## Supplementary Materials

The following supporting information can be downloaded at: https://www.mdpi.com/article/10.3390/cancers17233844/s1, Figure S1: Prognostic stratification of the risk score in stage-specific subgroups; Figure S2: Comparisons with similar studies indicate that our predictions hold greater predictive value; Figure S3: Original images from the Western Blotting experiment.

## Abbreviations

The following abbreviations are used in this manuscript:

LUAD	Lung adenocarcinoma
LADGs	LUAD-dependent genes
LASSO	Least Absolute Shrinkage and Selection Operator
HPA	Human Protein Atlas
TCGA	The Cancer Genome Atlas
GEO	Gene Expression Omnibus
