# Supplementary material for: Integrating Functional Genomic Screens and Multi-Omics Data to Construct a Prognostic Model for Lung Adenocarcinoma and Validating SPC25
Source: Cancers (Basel). 2025 Nov 29;17(23):3844. doi: 10.3390/cancers17233844 (PMC12691466; doi:10.3390/cancers17233844)
Supplement: Supplementary file 1 [file cancers-17-03844-s001.zip › Supplementary Material FigureS1.pdf]

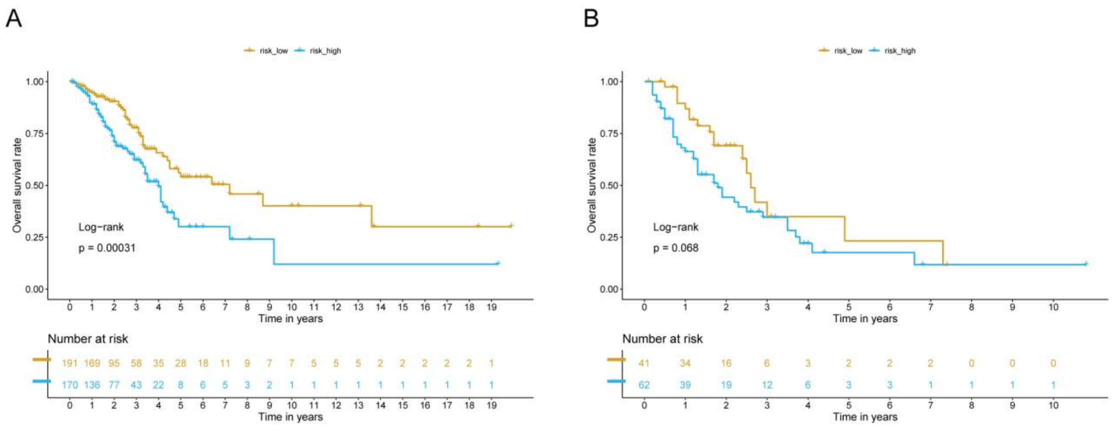

Prognostic stratification of the risk score in stage-specific subgroups.

(A) Kaplan-Meier curves for overall survival of early-stage (Stage I-II) LUAD patients in the high- and low-risk groups.

(B) Kaplan-Meier curves for advanced-stage (Stage III-IV) LUAD patients in the high- and low-risk groups.
