# Supplementary material for: Integrating Functional Genomic Screens and Multi-Omics Data to Construct a Prognostic Model for Lung Adenocarcinoma and Validating SPC25
Source: Cancers (Basel). 2025 Nov 29;17(23):3844. doi: 10.3390/cancers17233844 (PMC12691466; doi:10.3390/cancers17233844)
Supplement: Supplementary file 1 [file cancers-17-03844-s001.zip › Supplementary Material FigureS2.pdf]

<table>
<tr>
<td>Integrating Functional Genomic Screens and Multi-Omics Data to Construct a Prognostic Model for Lung Adenocarcinoma and Validating SPC25</td>
<td>Multi-omics data-based modeling reveals tumorigenesis- and prognosis-associated genes with clinical potential in lung adenocarcinoma(PMC12604227)</td>
<td>A 4-cuproptosis-related lncRNA theragnostic signature predicts survival and immunotherapy response in patients with lung adenocarcinoma(PMC12598532)</td>
<td>Identification and validation of prognostic genes for lung adenocarcinoma prognosis based on PANoptosis-related genes(PMC12540239)</td>
</tr>
<tr>
<td>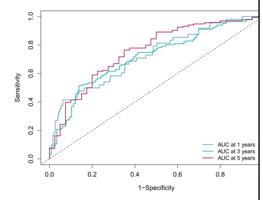
</td>
<td>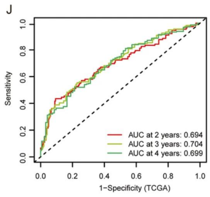
</td>
<td>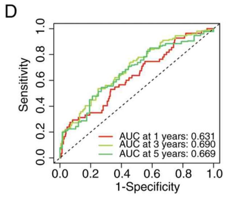
</td>
<td>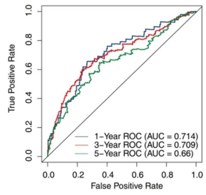
</td>
</tr>
<tr>
<td>Our model achieved AUC values of 0.714, 0.709, and 0.66 for predicting 1-year, 3-year, and 5-year OS in TCGA LUAD patients, respectively.</td>
<td>The model achieved AUC values of 0.694, 0.704, and 0.699 for predicting 2-year, 3-year, and 4-year OS in TCGA LUAD patients, respectively.</td>
<td>The model achieved AUC values of 0.631, 0.690, and 0.699 for predicting 1-year, 3-year, and 5-year OS in TCGA LUAD patients, respectively.</td>
<td>The model achieved AUC values of 0.714, 0.709, and 0.66 for predicting 1-year, 3-year, and 5-year OS in TCGA LUAD patients, respectively.</td>
</tr>
<tr>
<td colspan="4">In comparison with the other models, our model demonstrates significant advantages:
（1）Superior Short-Term Prediction: For the critical 1-year overall survival (OS) prediction, our model's AUC (0.714) is substantially higher than that of Model 3 (0.631), highlighting its exceptional capability for early risk stratification.
（2）Stable Mid-Term Prediction: For 3-year OS prediction, our model (AUC=0.709) also consistently outperforms both Model 2 and Model 3.
（3）0Comprehensive Time-Point Coverage: Unlike Model 2, our model provides predictions across the complete range from 1 to 5 years, making it more practical for a comprehensive assessment.
In conclusion, our model achieves the best overall performance in short- and mid-term predictions, establishing itself as a more reliable and comprehensive prognostic tool.</td>
</tr>
</table>
