# Supplementary material for: Integrating Functional Genomic Screens and Multi-Omics Data to Construct a Prognostic Model for Lung Adenocarcinoma and Validating SPC25
Source: Cancers (Basel). 2025 Nov 29;17(23):3844. doi: 10.3390/cancers17233844 (PMC12691466; doi:10.3390/cancers17233844)
Supplement: Supplementary file 1 [file cancers-17-03844-s001.zip › Supplementary Material FigureS3.pdf]

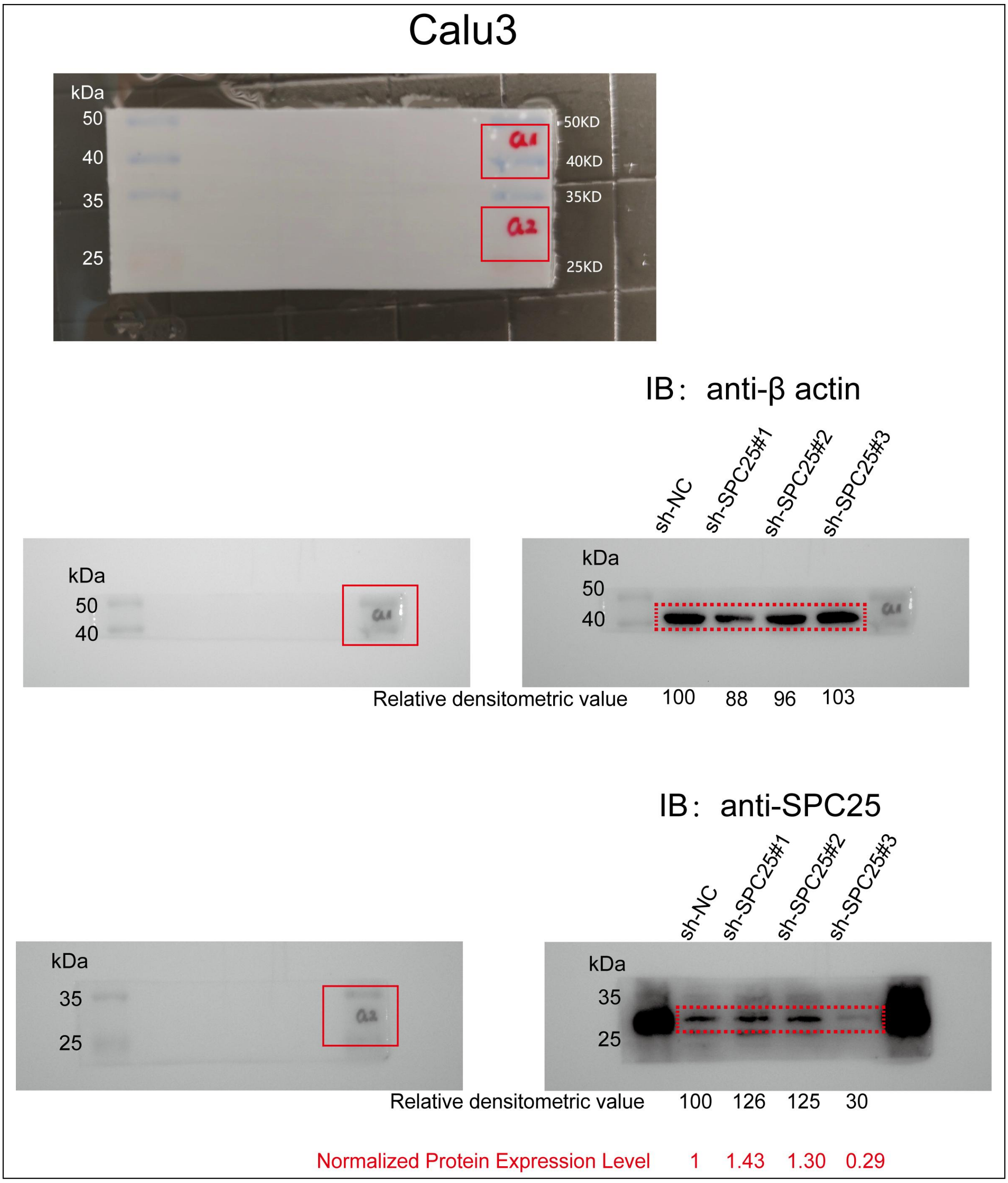
Calu3
kDa
50
40
35
25
50KD
40KD
35KD
25KD
IB： anti-β actin
sh-NC
sh-SPC25#1
sh-SPC25#2
sh-SPC25#3
kDa
50
40
Relative densitometric value 100 88 96 103
IB： anti-SPC25
sh-NC
sh-SPC25#1
sh-SPC25#2
sh-SPC25#3
kDa
35
25
Relative densitometric value 100 126 125 30
Normalized Protein Expression Level 1 1.43 1.30 0.29

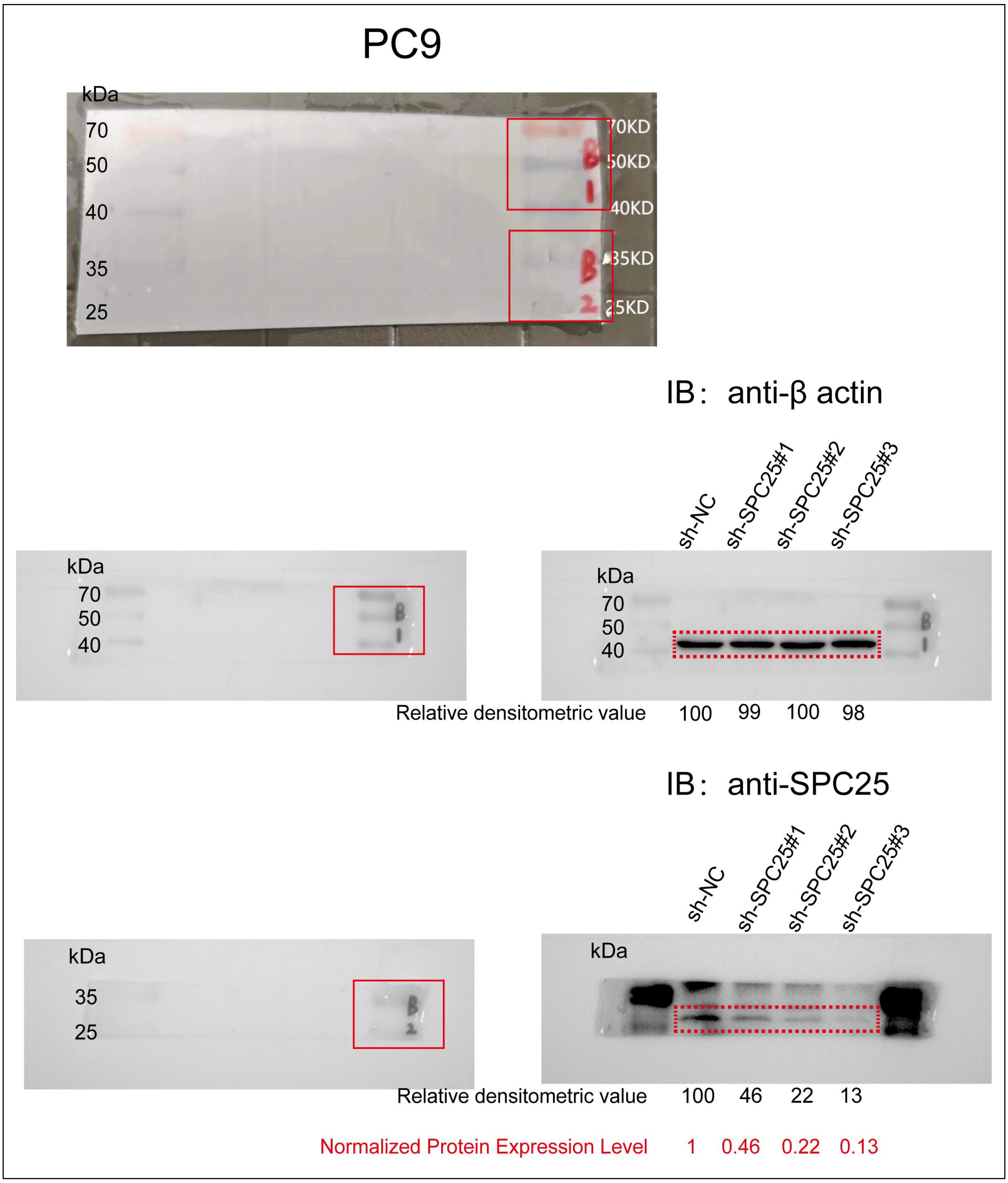
PC9
kDa
70
50
40
35
25
70KD
50KD
40KD
35KD
25KD
IB：anti-β actin
sh-NC
sh-SPC25#1
sh-SPC25#2
sh-SPC25#3
kDa
70
50
40
Relative densitometric value 100 99 100 98
IB：anti-SPC25
sh-NC
sh-SPC25#1
sh-SPC25#2
sh-SPC25#3
kDa
35
25
Relative densitometric value 100 46 22 13
Normalized Protein Expression Level 1 0.46 0.22 0.13
